# A Weld Defects Detection System Based on a Spectrometer

**DOI:** 10.3390/s90402851

**Published:** 2009-04-21

**Authors:** Daniel Bebiano, Sadek C. A. Alfaro

**Affiliations:** Automation and Control Group, University of Brasilia, Universidade de Brasília, Faculdade de Tecnologia, Departamento de Engenharia Mecânica, Brasília – DF, Brazil. CEP: 70910-900; E-Mail: dbebiano@unb.br

**Keywords:** GTAW, change detection, spectroscopy

## Abstract

Improved product quality and production methods, and decreased production costs are important objectives of industries. Welding processes are part of this goal. There are many studies about monitoring and controlling welding process. This work presents a non-intrusive on-line monitoriment system and some algorithms capable of detecting GTAW weld defects. Some experiments were made to simulate weld defects by disturbing the electric arc. The data comes from a spectrometer which captures perturbations on the electric arc by the radiation emission of chosen lines. Algorithms based on change detection methods are used to indicate the presence and localization of those defects.

## 1. Introduction

Welding processes are used in many manufacturing industries and due to this widespread application, many studies have been made to improve their quality. Some of these studies include visual inspections, destructive and non-destructive testing techniques, but these are only done after the process and this raises the final cost if defects are found and the products must be reworked or discarded.

There is some research that has studied the use of spectrometers as a welding sensor in Laser [1–2], GMAW [3–8] and GTAW [9–14] processes, including methodologies for defect detection [15] and control [2,16]. When a defect occur during the welding, it can disturb the weld bead, the magnetic field, electric field, weld temperature, weld sound, weld radiation emission and others factors. If a sensor is capable of detecting the perturbation, it is feasible to build a system based on this detection to monitor the weld quality.

This work proves that it is possible to improve a non-destructive and on-line weld defects monitoring system using the radiation emitted by chosen chemical elements, involved in the welding process, and measured by a spectrometer sensor. The main objectives are to evaluate if the spectrometer is capable of sensing perturbations in the electric arc and present algorithms able to point them out. Thus, it was not necessary, in this initial phase, to examine the defects metallurgically.

## 2. Plasma Spectroscopy

The physical phenomena consists of a photon emission at a determined wavelength or frequency after the absorption of some energy. This can be compared to a fingerprint. An atom and its ions and molecules can emit photons in different wavelengths, but a wavelength is related only to one atom or ion or molecule.

The study of plasma emission can be done in two different ways: qualitative and quantitative. In a qualitative approach, the analysis is made on the chemical elements found on the plasma. In a quantitative study, the objective is to evaluate some information extracted by the spectra taken. A common factor is the calculation of the plasma Electronic Temperature. Another that can be applied is the intensity of radiation emitted by some spectral lines. That one is proposed in this work.

## 3. Change Detection

The main idea of change detection techniques is to evaluate a signal and if there is an appreciative change in its behavior, in frequency, magnitude, abrupt peaks, the system must be capable of detecting them. These perturbations can be defects on the welding process.

In the case of this work, the process involved can be considered as a linear system in discrete time. The process equation is given in Equation (1) [17]:

(1) $$\theta_{t+1}=F_{t+1}\theta_{t}+w_{t}$$

where *t* is time instant; *F_t+1_* is the transition matrix from the state *θ_t_* to *θ_t+1_*; *θ*, in this work, is the radiation intensity; and *w* is the noise, represented as a random variable with normal distribution with zero mean and variance *Q*.

The model for the spectrometer reading is shown in Equation (2):

(2) $$y_{t}=\theta_{t}+v_{t}$$

**Figure 1.**
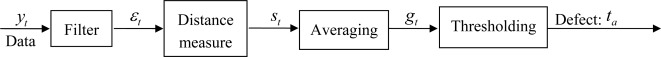
Change detection diagram flux.

The signal given by the sensor (*y_t_*) is the radiation emitted by the plasma (*θ_t_*) with an added noise (*ν_t_*). The noise also is a random variable with normal distribution with zero mean, but variance *R*. With this model it is presented a diagram flux of the change detection, given by Figure 1.

The sensor data (*y_t_*) is filtered. The filter estimates the radiation intensity (*θ_t_*). The residual (*ε_t_*) is calculated and, from this information, the distance measure is done. This value measures the difference between the sensor reading and the estimation given by the filter. The residual itself is an example of this distance. Then, it is done a statistic test (*g_t_*) based on the distance. Finally, *g_t_* is compared to a threshold (*h*) to decide if there is a defect in the weld. If the value is lower than the reference, it is assumed that the welding process is normal. But, if the statistical test value is greater than the threshold, it is possible that a defect occurred. There are many change detection algorithms. This work presents a widely used one, the Cusum LS Filter [16] and another is proposed, which applies steps from different algorithms.

The Cusum LS Filter uses a Least Square filter to estimate the radiation intensity, Equation (3). The distance is given by the residuals, Equation (4), and the statistical test is given by a cumulative sum, Equation (5). The factor *σ* is subtracted at each time instant *t* to avoid false alarms. Its value is chosen by the designer. And finally, the comparison of the statistic test to a threshold *h*, Equation (6). If its value is greater than *h*, an alarm is set and its instant, *t_a_*, is recorded, the statistics tests are reset and *t_0_* becomes *t*. With the values of the alarm instants, it is possible to indicate the defect’s position once the weld speed is constant:

(3) $${\hat{\theta }}_{t}=\frac{1}{t-t_{0}}\cdot \sum_{k=t_{0}+1}^{t}y_{k}$$

(4) $$\begin{array}{c} \varepsilon_{t}=y_{t}-{\hat{\theta }}_{t-1}\\ s_{t}^{\left(1\right)}=\varepsilon_{t}\\ s_{t}^{\left(2\right)}=-\varepsilon_{t} \end{array}$$

(5) $$\begin{array}{c} g_{t}^{\left(1\right)}=\text{max}\left(g_{t-1}^{\left(1\right)}+s_{t}^{\left(1\right)}-\sigma ,0\right)\\ g_{t}^{\left(2\right)}=\text{max}\left(g_{t-1}^{\left(2\right)}+s_{t}^{\left(2\right)}-\sigma ,0\right) \end{array}$$

(6) $$\begin{array}{lllll} \mathrm{if} & g_{t}^{\left(1\right)}>h & \mathrm{or} & g_{t}^{\left(2\right)}>h & \{\begin{array}{l} \mathrm{alarm}\;:t=t_{a}\\ g_{t}^{\left(1\right)}=g_{t}^{\left(2\right)}=0\\ t_{0}=t \end{array} \end{array}$$

**Figure 2.**
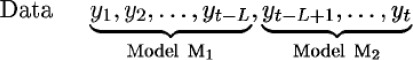
Scheme for sliding windows.

The other algorithm is based on sliding windows. The idea is to compare two models (two filters). For a better comprehension, a scheme can be seen in Figure 2. The slow filter, that estimates M1, uses data from a very large sliding window with size L. The fast filter estimates M2 by a small window. Then, two estimates, *θ̂*_1_ and *θ̂*_2_ with variances *P_1_* and *P_2_*, are obtained. If there is no abrupt change in the data, these estimates will be consistent. Otherwise, an alarm is set.

Both models present the notation of Equation (2). The filters for this change detection technique are also least square filters; i.e., they are Kalman Filters, Equations (7–8). One can see the filter for Model 1 in Equation (7) and the filter for Model 2, in Equation (8). The factor *K* is the filter gain and *P* is the covariance matrix. For the distance measurement the Brandt [18] algorithm, Equation (9), was chosen. The statistical test and thresholding are the same as presented before; they are shown in Equations (10) and (11), respectively:

(7) $$\begin{array}{l} \varepsilon_{t}^{(1)-}=y_{t-L}-{\hat{\theta }}_{t-1}^{\left(1\right)}\\ K_{t}^{(1)-}=\frac{P_{t-1}^{\left(1\right)}}{P_{t-1}^{\left(1\right)}-R}\\ {\hat{\theta }}_{t}^{(1)-}={\hat{\theta }}_{t-1}^{\left(1\right)}+K_{t}^{(1)-}\cdot \varepsilon_{t}^{(1)-}\\ P_{t}^{(1)-}=\left(1-K_{t}^{(1)-}\right)\cdot P_{t-1}^{\left(1\right)}+Q_{1} \end{array}$$

(8) $$\begin{array}{l} \varepsilon_{t}^{\left(2\right)}=y_{t}-{\hat{\theta }}_{t-1}^{\left(2\right)}\\ K_{t}^{\left(2\right)}=\frac{P_{t}^{\left(2\right)}}{P_{t}^{\left(2\right)}+R}\\ {\hat{\theta }}_{t}^{\left(2\right)}={\hat{\theta }}_{t-1}^{\left(2\right)}+K_{t}^{\left(2\right)}\cdot \varepsilon_{t}^{\left(2\right)}\\ P_{t}^{\left(2\right)}=\left(1-K_{t}^{\left(2\right)}\right)\cdot P_{t}^{\left(2\right)}+Q_{2} \end{array}$$

(9) $$s_{t}=\text{log}\left(\frac{R_{t}^{\left(1\right)}}{R_{t}^{\left(2\right)}}\right)+\frac{{\left(\varepsilon_{t}^{\left(1\right)}\right)}^{2}}{R_{t}^{\left(1\right)}+P^{\left(1\right)}}-\frac{{\left(\varepsilon_{t}^{\left(2\right)}\right)}^{2}}{R_{t}^{\left(2\right)}+P^{\left(2\right)}}$$

(10) $$\begin{array}{l} g_{t}^{\left(1\right)}=\text{max}\left(g_{t-1}^{\left(1\right)}+s_{t}^{\left(1\right)}-\sigma ,0\right)\\ g_{t}^{\left(2\right)}=\text{max}\left(g_{t-1}^{\left(2\right)}+s_{t}^{\left(2\right)}-\sigma ,0\right) \end{array}$$

(11) $$\begin{array}{lllll} \mathrm{if} & g_{t}^{\left(1\right)}>h & \mathrm{or} & g_{t}^{\left(2\right)}>h & \{\begin{array}{l} \mathrm{alarm}:t=t_{a}\\ g_{t}^{\left(1\right)}=g_{t}^{\left(2\right)}=0\\ t_{0}=t \end{array} \end{array}$$

## 4. Experimental Issues

**Figure 3.**
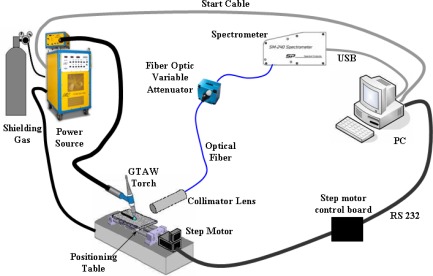
Experimental scheme.

Some experiments were performed using proposals of electrical arc perturbations taken from a bibliographic review [5–8]. First, we performed a study to evaluate the spectrometer as a sensor capable of detecting perturbations at the electrical arc. For this task, the experimental scheme seen in Figure 3 was set up. The power source is a multiprocessor welding machine. The weld torch had a tungsten electrode with 2% thorium and 1.6 mm diameter. The shielding gas was pure argon. The specimens were SAE 1020 steel with ⅛” width. The plate was moved by a positioning table. The motor responsible for its movement was a Berger Lahr model IDS91 controlled by a board with a microcontroller. The data acquisition and the weld machine were controlled by a computer equipped with Labview software. The spectrometer was a Spectral Products model SM 240-USB. In all experiments, the spectrum taken was a three sample mean with 2 ms of integration time. The area focused on the plasma was between the electrode and the plate but not including them. A system with collimator lenses was responsible for focusing the lecture and transmitting to an optical fiber of Ocean Optics, code P200-5-UV/VIS. The light had its intensity attenuated by an Ocean Optics, model FVA-UV device to avoid signal saturation.

Four different kinds of defects were induced to evaluate the capacity of the sensor to detect their perturbation on the electrical arc. One of them was a metallic inclusion. It consists on small pieces of welding wire. Another defect was simulated by putting some sand, which is used for cooling welded parts, on the welding track. Another disturbance applied was on the gas flow rate, which was changed from 10 to 2 L/min. Finally, some water was sprayed at the electrical arc.

The software acquired the spectral graph and saved it after the experiment. With these data, a quantitative analysis of some spectral lines was made. Several experiments were done to study the wavelengths’ behavior with different weld parameters. Initially, two elements were selected: iron, found in the plates; and hydrogen, which can arise from contaminations. Some initial wavelengths were chosen of those elements and their corresponding ions based on the NIST [19] tables. It was observed that for different current values the lines behaved differently, especially for iron. For example, one observed line at 120 A presented fluctuations in the disturbed region, but remained constant at 180 A for the same defect perturbations. The chosen lines were, for iron, 403.5 nm and 487.8 nm; and for hydrogen, 656.3 nm. To analyze the experiments, a Matlab file was developed. For each spectral line its signal was filtered, its residual calculated, and the distance and the statistical test further compared to the threshold.

## 5. Results and Discussion

**Figure 4.**
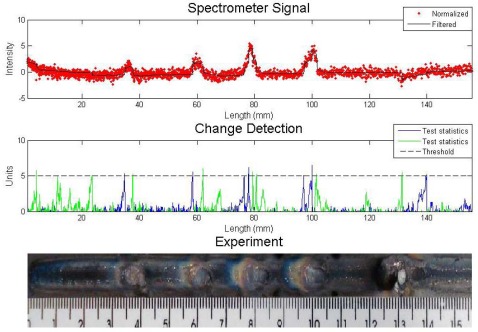
Experiment with metallic and sand inclusions – analysis with Cusum LS Filter.

An experiment with metallic and sand inclusions is presented in Figure 4. The algorithm for defect detection was the Cusum LS Filter with *σ* = 0.5 and *h* = 5. The values are arbitrary and they are set to result in fewer errors, like false detection or lack of detection. One can see in the first graph the sensor reading and the result of the filter. The signal was normalized since the study is interested in the signal fluctuations, not its absolute value. The spectral line chosen was iron at 487.8 nm. Welding parameters were current 90 A, stand off 7 mm, welding speed of 2.5 mm/s and gas flow rate 10 L/min.

The four metallic inclusions were detected with a great fluctuation due to the increase of iron in the arc region. There is a disturbance where the sand was included (last defect). There was no iron increase because the sand does not contain this chemical element in its composition, but the sand caused an interference in the electric arc reflected by a fluctuation of all spectral lines.

**Figure 5.**
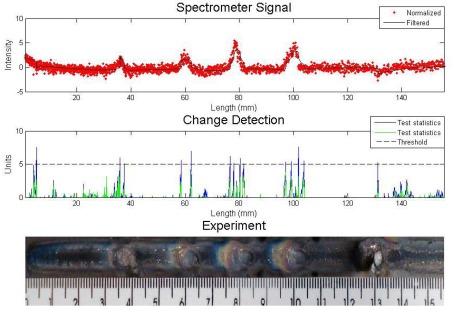
Experiment with metallic and sand inclusions – analysis with proposed algorithm.

The same experiment was analyzed by the algorithm proposed with the same algorithm input parameters: *σ* = 0.5 and *h* = 5. The spectral line was also the same. The result can be seen in Figure 5. The four metallic inclusions and the sand inclusion were also detected. The difference between the performances is found in the statistic tests. In the proposed algorithm, the value was near zero when there were no apparent defects and was high along the defect. The Cusum LS Filter presented a higher value of statistic test when there were no apparent defects.

**Figure 6.**
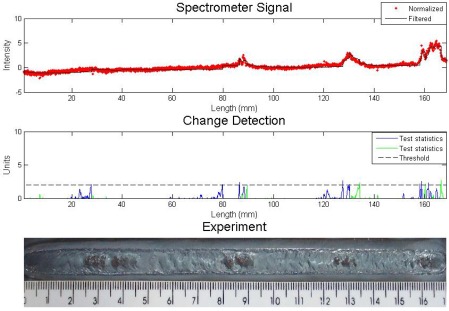
Experiment with shielding gas disturbance – analysis with Cusum LS filter algorithm.

**Figure 7.**
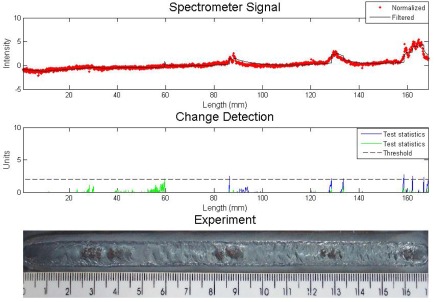
Experiment with shielding gas disturbance – analysis with proposed algorithm.

The result of the experiment which had the gas flow rate changed four times from 10 to 2 L/min can be seen in Figure 6 with Cusum LS Filter analysis and in Figure 7 with the other algorithm.

The experiment welding parameters were current 160 A, stand off 5 mm and welding speed of 2.5 mm/s. The algorithm parameters were both the same: *σ* = 0.5 and *h* = 2. The spectral line chosen was iron 403.5 nm. The statistical test of the first algorithm exceeded the threshold value in each change in the gas flow rate, but this did not happen with the proposed algorithm, which missed the first defect. One can see in the filter sign, that the variations were almost imperceptible for both analyses.

**Figure 8.**
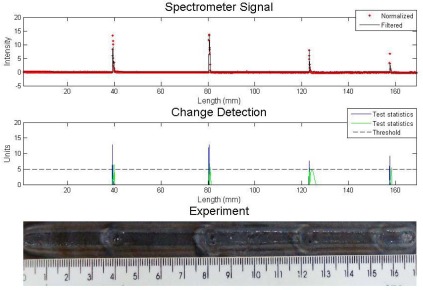
Experiment with sprayed water – analysis with Cusum LS Filter.

**Figure 9.**
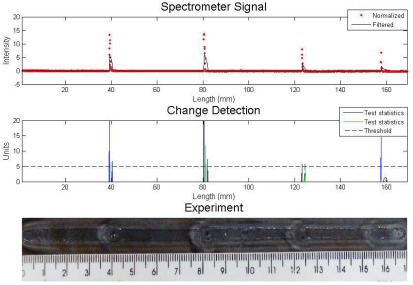
Experiment with sprayed water – analysis with proposed algorithm.

In Figures 8 and 9 one can see the result of the experiment that consisted of spraying water at the electrical arc. Welding parameters were current 90 A, stand-off 5 mm, shielding gas at 10 L/min and welding speed of 2.5 mm/s. Both algorithm input variables were *σ* = 0.5 and *h* = 5. The spectral line chosen was hydrogen, 656.3 nm.

Both figures presents equivalent outcomes. All four sprays were detected. One difference can be found on how high the statistical test values were in the proposed algorithm compared to the Cusum LS Filter.

**Figure 10.**
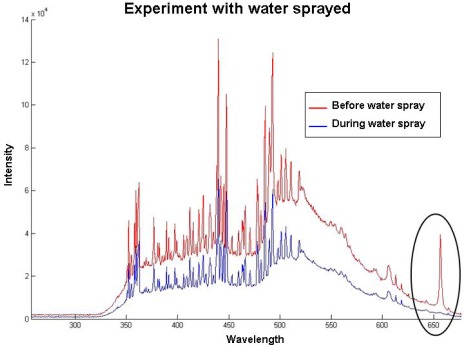
Spectrum before and during water spray.

Figure 10 presents the full spectrum observed before and during the water spray. One can notice the high peak in the hydrogen line due to its presence in the electric arc region. The spectrum represented by red line –during water insertion- was multiplied by a factor near 1 to differentiate the two spectra.

In all experiments, other welding parameters or input algorithm variables and spectral lines can be used. Different values were tested and lead to similar results, especially the algorithm parameters, which depend on the designer experience.

It is possible to capture the spectrometer signal and run the algorithms in real time. It can be done with dedicated hardware for data acquisition and processing the information. This task can be done by a microprocessor or FPGA technology. The algorithms will be modified in their input parameters, when the signal would not be normalized, but filtered in real time.

## 6. Conclusions

This work presented a system based on a spectrometer and change detection algorithms that could detect disturbances in an electric arc. These perturbations could be related to weld defects. Thus, it is possible to apply those techniques to monitor the weld quality. The Cusum LS Filter and the proposed algorithm were capable in pointing out simulated defects in the signal acquired by the sensor. Therefore, they can be applied in monitoring the weld quality. There are many possibilities of using different spectral lines, algorithms, and input parameters to capture disturbances in the spectrometer signal.

There was no need in calculating the Electronic Temperature. In this work, the information monitored was the radiation emitted by the electric arc. Thus, the computational cost is lower and the defect detection is faster. The algorithms can be applied in real time, once there is a test for each time instant, i. e., for each data acquired. For this purpose, it would be better to build some dedicated hardware for this task.
